# The Pivotal Role of the Gut Microbiome in Colorectal Cancer

**DOI:** 10.3390/biology11111642

**Published:** 2022-11-09

**Authors:** Ruqaiyyah Siddiqui, Anania Boghossian, Ahmad M. Alharbi, Hasan Alfahemi, Naveed Ahmed Khan

**Affiliations:** 1 College of Arts and Sciences, American University of Sharjah, Sharjah 26666, United Arab Emirates; 2 Department of Medical Biology, Faculty of Medicine, Istinye University, 34010 Istanbul, Turkey; 3 Department of Clinical Laboratory Sciences, College of Applied Medical Sciences, Taif University, Taif 21944, Saudi Arabia; 4 Department of Medical Microbiology, Faculty of Medicine, Al-Baha University, Al-Baha 65799, Saudi Arabia; 5 Department of Clinical Sciences, College of Medicine, University of Sharjah, Sharjah 27272, United Arab Emirates

**Keywords:** colorectal cancer, microbiome, dysbiosis, fecal microbiota transfer, postbiotics, prebiotics

## Abstract

**Simple Summary:**

Colorectal cancer is a common form of cancer observed globally. It is thought that the gut microbiome may play a pivotal role in the development and progression of colorectal cancer in patients. Furthermore, current treatment strategies may lead to a variety of side effects, and chemotherapeutic resistance is observed. Consequently, new types of treatments should be considered, including post/pre/synbiotics and fecal microbiota transfer, which may be able to restore gut microbial dysbiosis.

**Abstract:**

Colorectal cancer is the third most diagnosed cancer worldwide and the second most prevalent cause of cancer-related mortality. It is believed that alterations within the gut microbiome may impact the development and progression of cancer. Additionally, the diet an individual maintains and the amount of alcohol consumed can alter the microbiome, thus impacting the development of colorectal cancer. A diet focused on fiber intake is considered beneficial, as it contains short-chain fatty acids such as butyrate, which have antitumor properties. Furthermore, current treatment strategies, such as chemotherapy, have various side effects. In this review, we discuss the role of the gut microbiome and oral bacteria in relation to colorectal cancer. We also deliberate on the role of diet and alcohol consumption in the development of colorectal cancer. Moreover, the influence of the various metabolites within the gut and the importance of gut inflammation in the development of colorectal cancer are explained. Finally, potential therapies such as fecal microbiota transfer and post/prebiotics are elaborated on. To further comprehend risk factors in the development of colorectal cancer, future studies are warranted to determine the precise mechanisms of action between the gut microbiome and carcinogenesis in order to develop therapies that may target gut microbial dysbiosis.

## 1. Introduction

Cancer is the most common disease impacting individuals; it can affect one in two people in industrialized countries. Additionally, it has continued to be a burden upon societies worldwide [1]. Furthermore, the most prevalent forms of cancers are those occurring within the gastrointestinal tract [2,3]. Gastrointestinal cancers are the various cancers arising within the gastrointestinal tract or in any of its associated organs, the most prevalent of which are: the stomach, liver, pancreas, esophagus, and colorectal cancers [3]. Importantly, hepatocellular carcinoma (HCC) is believed to be one of the leading causes of cancer-induced deaths globally [4]. Furthermore, in the case of colorectal cancer (CRC), one in three people are expected not to live past five years once diagnosed [5]. Of various cancer-related mortalities, CRC is the second most common in the United States; this form of cancer may develop from the proximal colon, distal colon, or rectum. Hence, it can also be referred to as bowel cancer or rectal cancer. Interestingly, 41% of CRC develops in the proximal region of the colon, 28% in the rectum, and 22% in the distal colon [5,6,7,8]. Additionally, high rates of CRC-diseased individuals are found in New Zealand, Europe, Australia, and North America [8]. Moreover, according to the Global Cancer Observatory, 10.2% of diagnosed tumors and 9.2% of deaths were due to colorectal cancer [9]. In fact, by the year 2030, the rate of CRC incidence is predicted to increase by 60% and become a global burden [5,7,10].

One of the major complex networks in the human body is the gut, as it hosts a variety of microorganisms, including bacteria [1,11]. It is believed that an individual’s gut microbiome may impact the development of cancer; an individual with an imbalanced gut microbiome, a condition referred to as dysbiosis, may be more susceptible to the development of CRC [1,12]. Additionally, the diet maintained and the amount of alcohol consumed by an individual may alter their microbiome, thus increasing their susceptibility to CRC [13,14]. In this review, the relationship between CRC and the gut microbiome is discussed, as well as the relationship between an individual’s diet and alcohol consumption towards their risk of developing CRC. Furthermore, the alterations in the metabolites within the gut, as well as the inflammation of the gut, are elaborated on. Finally, potential treatments for CRC such as fecal microbiota transfer, postbiotics, prebiotics, and synbiotics, are discussed.

## 2. What Is Colorectal Cancer

Colorectal cancer is considered to be the third most commonly diagnosed form of cancer [15]. It is the second most common form of cancer in women and the third most common in men [16,17]. Furthermore, CRC has the second deadliest malignancy for both genders together [15]. In simpler terms, colorectal cancer is the second leading cause of cancer death worldwide [16]. Often at times, this disease is thought of as being a so-called westernized disease, with the highest number of cases being in Australia, New Zealand, North America, and Europe [16]. Although there may be potential differences in the site of origin depending on age and gender, 41% of CRC occurs in the proximal colon, 22% involves the distal colon, and 28% involves the rectum [6].

The food consumption of an individual affects their chances of developing CRC, along with other factors. For example, consuming processed and unprocessed meat leads to an increase in their risk of obtaining CRC; however, that risk is reduced through fiber consumption [16]. Nevertheless, the consumption of processed or unprocessed meat is not the only factor influencing the chances that an individual may obtain colorectal cancer. In fact, colorectal carcinogenesis is a heterogeneous process that is influenced by various factors such as diet, microbial and environmental exposures, and host immunity [16]. For this reason, a person maintaining a high-meat, high-fat, low-fiber diet will not necessarily get colorectal cancer [16]. Similarly, an individual maintaining a healthy diet rich in fruits and vegetables will not be protected from the disease. However, this does not decrease the important role an individual’s diet plays in CRC. The diet an individual maintains plays a major role in the initiation, promotion, and progression of the neoplastic process [16]. The neoplastic process refers to the accumulation of somatic mutations in specific genes that ultimately give rise to tumor cells [18]. Hence, it is believed that colorectal cancer follows a stepwise disturbance pattern resulting in its arousal. This disturbance pattern involves disturbances in the gut microbiota, which may be induced by components found in the diet of an individual, in addition to genetic alterations in tumor-suppressor genes and oncogenes [16]. The gut microbiota may have the ability to influence the development of colorectal cancer in several ways.

## 3. Gut Microbiome and Its’ Influence on the Colon

As mentioned earlier, one of the key factors playing a role in colorectal carcinogenesis is the environment of the gut microbiome. The gut microbiome constitutes a rich and diverse community of microorganisms [11]. This ecosystem is formed before birth and develops to become a fully functioning and stable microbiome within 2 to 3 years [19,20]. The human intestine is estimated to contain more than 2000 microbial species [12]. In addition, the most heavily microbial colonized section of the digestive system is the colon. It is estimated to contain around 70% of the human microbiome [11].

These microbial species perform a variety of functions, some of which include metabolizing indigestible food, modulating immune response, and synthesizing nutrients [12]. Moreover, it is now evident that the process of acquiring and maintaining gut microbes is fundamental for an individual’s health [21]. These microbes are vital in the formation of mucosal immunity [22]. For example, a class of microbicidal proteins in Paneth cells known as angiogenin-4 can be secreted against microbes into the gut lumen [22]. The commensal bacteria residing within the intestine are capable of enhancing the intestines’ innate immunity by modulating toll-like receptors (TLRs) expression on immune cells’ surface via pathogen-associated molecular patterns leading to the expression of antimicrobial peptides (AMPs) [23]. Microbes lead to the activation of T-cells by activating the nuclear factor-kappa B signaling pathway, which in turn leads to the stimulation of cytokine production and overexpression of costimulatory molecules on the antigen-presenting cells (APCs) [23]. In turn, the TLRs activation leads to the induction of islet-derived protein 3 gamma (Reg3g) expression [23]. The TLR activation induces the inhibition of inflammatory action contributing to intestinal homeostasis [23].

Additionally, the microbes found within the gastrointestinal tract are capable of communicating with each other, as well as with the host [21]. This communication feature may ultimately result in great effects on disease and health development [21]. In addition to immune response, this communication is also essential for appropriate mucosal function [19]. Furthermore, this crosstalk is mediated by metabolites, proteins, and small RNAs [19]. These communicate together via epithelium [19]. In this regard, since the gut microbiome interacts with the host, it contributes to the process of carcinogenesis [11]. Furthermore, the colon is thought to be the most disposed to cancer development upon its comparison to other sections of the digestive tract [11].

Alterations in the gut microbiome may contribute to various diseases. This may be because of their role in metabolism and immune function [12]. These alterations are known as dysbiosis and are facilitated through changes in the Mus musculus miRNA [19]. For example, changes occurring within the intestinal microbiome may result in the initiation and promotion of colorectal cancer [12]. *Fusobacterium nucleatum* and *Escherichia coli*, through the uptake of specific human sncRNA, regulate the expression of microbial genes, thus affecting their growth [19]. *Fusobacterium nucleatum* is the most found gut bacterium in CRC patients; this bacterium is a gram-negative anaerobe [24]. Furthermore, this bacterium acts as a prognostic biomarker; at higher levels it usually means a shorter overall survival [24].

**Figure 1:**
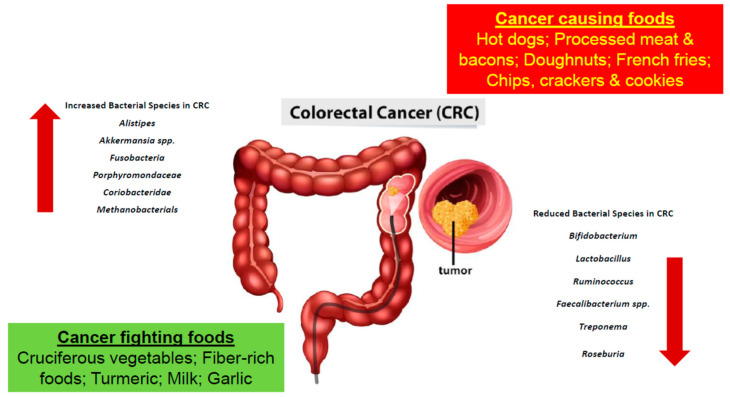
The development of colorectal cancer is a result of a multifactorial process involving the consumption of various foods and gut microbial dysbiosis.

Furthermore, based on studies obtained, certain bacteria are found to be greater in number in CRC patients, while others are found to lessen [16,24]. Bacteria such as *Alistipes*, *Akkermansia* spp. *Fusobacteria*, *Porphyromonadaceae*, *Coriobacteridae*, and Methanobacteriales were found to be increasing in number in the colon microbiota of a CRC patient [16,25]. More specifically, *Bacteroides fragilis*, *Escherichia coli*, *Fusobacterium nucleatum*, *Enterococcus faecalis,* and *Streptococcus gallolyticus* were all linked to CRC [26]. In a study conducted, an increase in polyketide synthase pks island-positive *Escherichia coli* was found in the colon tissues isolated from CRC patients [27]. Additionally, in another study conducted with CRC patients, *F. nucleatum*, an oral bacterium, which will be elaborated on further in the upcoming sections, was also found within the colorectal tumors of the patients [27]. Furthermore, in another study conducted on mice, tumor-bearing mice were found to have an increase in the number of Bacteroides within their fecal samples [28]. Moreover, bacteria such as *Bifidobacterium*, *Lactobacillus*, *Ruminococcus*, *Faecalibacterium* spp., *Treponema*, and *Roseburia* were decreasing in number (Table 1 and Figure 1) [16,25]. Further studies were performed using germ-free mice. The fecal samples of patients with CRC and healthy individuals were transplanted into the mice in which cancer was promoted chemically. It was recorded that the rate upon which the tumor was generated was associated.

With microbial composition. Gram-positive bacteria such as Clostridium group XIVa were found to associate negatively with tumor generation. In contrast, Gram negative bacteria such as *Alistipes, Akkermansia*, *Parabacteroides*, and *Bacteroides* are associated positively with tumor generation [16,25,29].

Moreover, through evidence obtained, it is proven that alterations in the gut microbiome occur during the initial stages of colorectal carcinogenesis [12]. It is hypothesized that alterations in colonic flora may create a more favorable microenvironment for tumor development [11]. Bacterial micro vesicles may play a role in tumorigenesis, and in fact, their role is underestimated [19]. There is a possibility that the extracellular vesicles from the host and microbiota in the intestinal ecosystem promote tumor survival and multi-drug resistance [19]. Furthermore, with changes in the gut microbiota, it may be possible to identify the precursor lesion for CRC: colorectal adenoma, for individuals at risk [12]. It may be possible to modify the intestinal microbiome to aid in the prevention of CRC [12].

## 4. Oral Bacteria and Its Role in Colorectal Cancer

The discovery of oral bacteria dates back to the 1670s, when Antony Van Leeuwenhoek reported the presence of various microbes within the plaque on tooth surfaces, since then to examine multispecies microbial communities, researchers have studied the human oral microbiome [30,31]. The oral microbiome is a complex ecosystem containing billions of bacteria, with approximately 700 predominant taxa [32,33]. The bacterial taxa colonizing the oral cavity contribute to oral health and oral diseases [33]. The various bacteria present create their own balanced ecosystem in which they are capable of surviving [32].

Oral bacteria have been shown through molecular methods to be involved in colorectal cancer [19]. In fact, through various studies, it has been observed that various oral bacteria may play an essential role in the development of colorectal cancer [34]. Tissue samples from the intestinal mucosa have been collected from CRC patients, in which higher numbers of *Fusobacterium*, *Peptostreptococcus*, *Mogibacterium* spp., and *Porphyromonas* have been found (Table 2) [32]. Additionally, in another study, fecal samples were collected, and an increase in oral bacterial species was found. Those bacteria include *Actinomyces*, *Corynebacterium*, *Mogibacterium*, *Haemophilus*, and *Porphyromonas* [32].

Furthermore, oral bacterial species such as *Fusobacterium* and Bacteroides fragilis are found in both primary and metastatic CRC in humans [19]. A high abundance of the *Fusobacterium* is believed to be associated with tumor location and regional lymph node metastases [34]. Moreover, a type of *Fusobacterium* known as *Fusobacterium nucleatum* is of specific interest since it has only recently been linked to colorectal cancer [35]. Associations between the gram-negative *F. nucleatum* bacteria and colorectal cancer in humans have been found in patients during the different disease stages [35].

As mentioned earlier, this bacterium’s role has only recently been discovered hence why its role as a cancer-causing microbiota is still emerging; with new revelations being found regarding its various contributions to the development, growth, and spreading of cancer [35]. Moreover, the nucleic acids of *F. nucleatum* present in CRC tissues have been studied; this bacterium was found to be present within the tissues using the various molecular approaches, including 16 s ribosomal RNA (rRNA) gene amplicon sequencing, RNA sequencing (RNA-seq), directed quantitative PCR (qPCR) and DNA sequencing (DNA-seq) [35]. Further studies to elucidate the precise mechanism of the role of this bacterium in the development of CRC are thus warranted.

## 5. The Relationship between an Individual’s Diet and Colorectal Cancer

An individual’s lifestyle, as well as the food they consume, can increase or decrease their chances of being diagnosed with colorectal cancer. As dietary patterns are changing in developed countries, individuals are increasing in weight and heading towards the obese and overweight sections of the scale [36]. Furthermore, overweight individuals whose body mass index (BMI) is between 25 kg/m^2^ to 29.9 kg/m^2^ are 19% more likely to be diagnosed with CRC compared to individuals whose BMI is between 20 kg/m^2^ to 25 kg/m^2^ [36]. Studies have shown that certain diets may tend to increase the risk of CRC, while others, such as the Mediterranean diet, tend to decrease it [13].

As mentioned earlier, due to the changing dietary patterns in developed countries, and the increase in consumption of red meat, there is an increase in CRC cases. In fact, approximately 60% of CRC cases occurring in developed countries are due to the unhealthy diet and lifestyles being followed [13]. Individuals that are maintaining a diet rich in processed or red meat and high-fat dairy products, as well as various fast foods and drinks, are more prone to being diagnosed with CRC [36]. Furthermore, with an increase in fat intake, there is a higher secondary bile production as well as insulin resistance which leads to the facilitation of carcinogenesis [36]. With this increase in fat intake and the maintenance of an animal-based diet, there is a greater number of bile-tolerant microorganisms such as *Alistipes*, *Bacteroides*, and *Bilophila* (Table 3) [37].

Moreover, maintaining a diet rich in fiber is important as well. In fact, individuals sustaining a diet rich in whole-grain consumption were found to have a reduction in their chances of developing CRC [39]. The term “dietary fiber” is a broad term referring to carbohydrate polymers consisting of ten or more monomeric units which the small intestine can neither digest nor absorb [38]. Although they are incapable of being digested by the small intestine, dietary fibers are very beneficial. They have been proven to affect the metabolic activities in an individual’s gastrointestinal tract [39]. It was found that the relation between an individual’s fiber intake density and their risk of developing CRC was inversely proportional. In addition, a high intake of dietary fiber is also associated with higher survival rates [39]. Furthermore, the fermentation of certain fibers to short-chain fatty acids plays a major role in CRC prevention. The importance of short-fatty acids will be further explained in the coming paragraphs.

As stated earlier, the diet an individual follows has a great influence on their gut microbiota. For example, one diet may have the ability to promote the growth of certain bacterial species, which in turn may alter various processes such as fermentative metabolism [37]. This alteration will lead to changes in an individual’s intestinal pH, thus increasing the chances of the development of pathogenic flora [37]. To further elaborate, individuals with high fat are not only more prone to CRC, but the diet these individuals follow may lead to the promotion of pro-inflammatory gut microbiota [37]. As a result, the intestine becomes more permeable, and pathogenic bacteria can take over. To further understand what is meant when stating that any type of alteration to the gut microbiota can lead to various gut-microbiota diseases, the changes (diet-depended) in the microorganisms of the gut-microbiota is going to be further elaborated [37].

Initially, of the complete microbiota, four major microbial phyla are believed to represent more than 90% of the bacteria present in the gut. These phyla are *Firmicutes*, *Bacteroides*, *Proteobacteria,* and *Actinobacteria* [4,37]. Furthermore, the gut microbiota has three enterotypes. The word enterotype refers to the stratification of the human gut microbiota, which serves to reduce a large number of global microbiome variations into just a couple of categories; this term first appeared in Nature back in 2011 [40]. Within the three enterotypes, there is a specific group of bacteria that is more abundant when compared to the others. For example, in enterotype 1, Bacteroides are more abundant, while in enterotypes 2 and 3, *Prevotella* and *Ruminococcus*, are more prevalent, respectively [37]. When an individual pursues a high-fat and protein diet, the growth of bacteria within enterotypes 1 and 3 are enhanced, compared to an individual pursuing a diet rich in carbohydrates whose enterotype 2 would be increased [37,41].

Moreover, studies were conducted to understand the changes in the gut microbiota when different diets are followed. The gut microbiota of African and European children was studied [37]. The African children came from rural Africa, whereas the Italian children came from urban areas [37]. Based on this study, it was found that the African children contained more Bacteroidetes, whereas the European children contained more *Enterobacteriaceae* [37]. Furthermore, the high consumption of red meat and low-fiber food, a form of diet referred to as the “Western diet,” is believed to cause an increase in the number of bacteria in the *Bacteroides* phyla and *Ruminococcus* [37]. Basically, a high-fat diet means a prevalence of *Bacteroides* and *Actinobacteria*, while a high fiber intake means less of *Bacteroides* and *Actinobacteria* [37]. On the other hand, a high fiber intake means an increase in *Firmicutes* and *Proteobacteria* [37]. Bacteroides-prevalent enterotype is associated with animal fats and proteins, while Prevotella-led enterotypes are associated with the high consumption of sugars and carbohydrates [37].

Additionally, individuals maintaining a diet rich in fruits and vegetables, as well as whole grain cereals, white meat, and fish, are less likely to be diagnosed with CRC [36]. In fact, individuals living along the Mediterranean coast follow a Mediterranean diet, which has proven to show a decrease in cancer mortality rates [13]. Olive oil, red grapes, and tomatoes are three constituents of the diet that have been proven to reduce the risks of CRC [13]. Olive oil, the very center of the Mediterranean diet, is a polyphenol that is believed to reduce the risk for CRC [13]. This polyphenol is believed to contain many chemopreventive effects since it interferes with the initiation, promotion, and progression of the cancerogenesis pathway [13]. Furthermore, phenolic derivatives generally tend to contribute to the cell adhesion processes, as well as tumor angiogenesis and migration [36]. Red grapes contain resveratrol on their external skin, which is a phenolic compound that is found mainly in red wine [13]. This compound contains various pharmacologic properties, including affecting the number of molecular targets of different cancer types [13]. Furthermore, this compound has the capability to deregulate the multiple pathways which affect cancer cell growth as well as oncogenic signaling [13]. Finally, of the many benefits tomatoes possess, one of them may be the prevention of cancer [13]. An individual who consumes tomatoes daily has a 20% decrease in their risk of obtaining CRC [13]. The reason tomatoes may have such a tremendous effect on CRC is possibly due to their high level of carotenoids, especially Beta-carotene and lycopene [13]. However, prospective studies are needed to comprehend the precise role of these carotenoids.

## 6. The Effects of Alcohol on the Gut Microbiome and CRC

On a global scale, excessive alcohol drinking is an issue on both pathological and financial scales, potentially leading to the death or disability of an individual [42,43]. With almost 2 billion people consuming alcohol on a daily basis worldwide, alcohol consumption has been listed as the fifth leading risk factor for disability and premature death of individuals between the age range 15 and 49 [42,44]. Additionally, alcohol consumption is also associated with organ dysfunction and tissue injury, as well as an increased risk of the development of cancer, specifically gastrointestinal cancers, which may occur in the liver, stomach, esophagus, oral cavity, rectum, and colon [14]. Moreover, alcohol consumption leads to changes in the composition of the gut microbiota and its functions; alcohol fed mice showed a decrease in good bacteria and an increase in endotoxin-producing bacteria [14,42,43,44,45].

As mentioned earlier, the gut microbiota is a diverse environment acting as a microbial reservoir within which thousands of different species, including bacteria, exist; most of the bacteria present belong to the Gram-positive phyla of *Firmicutes* or Gram-negative *Bacteroidetes* [42,44]. Additionally, earlier, dysbiosis was defined as the disruption or alteration of the intestinal microbiota. In addition to the various factors which can lead to dysbiosis, alcohol consumption is one [43]. Dysbiosis leads to various diseases, such as alcohol-related liver disorders, cancer, irritable bowel syndrome, and much more [44].

Various studies were conducted to understand the effect alcohol consumption has on the gut microbiome [42]. Using mice and rats, bacterial overgrowth and dysbiosis were induced through alcohol consumption [42]. For a period of 3 weeks, C57BL/6 mice were intragastrically fed alcohol, making up 40% of their total calories [42]. Furthermore, the control group was intragastrically fed an isocaloric diet. Based on this study, the alcohol-fed mice were found to have developed the alcoholic liver disease (ALD) [42]. ALD is a disease associated with bacterial overgrowth and dysbiosis within the start of the large intestine, a region also known as the cecum [42]. An increase in the number of *Bacteroidetes* and *Verrucomicrobia*, while a decrease in the number of *Firmicutes* was found in alcohol-fed mice. Whereas, in the control group *Firmicutes* was predominant (Table 2) [42].

As previously stated, CRC is attributed to various factors, such as red meat consumption and lack of fiber intake; however, another major risk factor is the consumption of alcohol [14]. Based on data obtained from the Korean Multi-Center Cancer Cohort study, men with a greater history of alcohol consumption are at greater risk of CRC. Additionally, it has been recorded by the American Institute for Cancer Research and the World Cancer Research Fund that 30 g/day of ethanol consumption could lead to CRC [14]. Gender, age, body weight, and ethnicity are all factors which influence the absorption, disposition, and metabolism of alcohol/ethanol. Using alcohol dehydrogenase (ADH), catalase, or cytochrome P450 2E1 (CYP2E1), ethanol is metabolized to acetaldehyde which is then oxidized to aldehyde dehydrogenase (ALDH) [14]. Unfortunately, acetaldehyde, which is known to induce DNA damage in the digestive tract, is classified as a Group 1 carcinogen to humans by the International Agency for Research on Cancer [14]. Acetaldehyde levels in the colon and stomach are dependent and influenced by the various microbes colonizing the colon as well as the genes responsible for coding alcohol-metabolizing enzymes such as ALDH2 [14].

In addition to metabolism by ADH, ethanol is further metabolized through oxidative metabolism to yield acetaldehyde. However, within the colonic mucosa, there is low ALDH activity; hence, significant amounts of acetaldehyde accumulate in the colon resulting in CRC [14]. Additionally, excessive alcohol consumption leads to an excessive intake of ethanol which alters the composition of the microflora and leads to an overgrowth of gram-negative bacteria; hence, the intestinal epithelial barrier will be disrupted. As a result, the barrier is now more permeable, and an increase in the accumulation of proinflammatory cytokines is found. Moreover, alcohol, when consumed in large amounts, leads to the promotion of inflammations [45]. Ethanol also leads to inflammatory reactions in the colonic mucosa and submucosa [45]. The neutrophil markers myeloperoxidase and granulocyte receptor-1 were found in ethanol-fed AOM-DSS mice. Furthermore, with ethanol administration, the expression of proinflammatory cytokines and chemokines significantly increased [45].

## 7. The Metabolites Present in the Gut Microbiome

As addressed earlier, the gut microbiota is speculated to have a major role in the susceptibility and progression of CRC [39]. Only recently, it has been shown that an important factor responsible for the connection of the intestinal microbiota to CRC is microbial metabolites [46]. These metabolites are believed to play a major role in tumor repressive functions [46]. Along with the occurrence of dysbiosis, which is a major characterization of CRC, the altered production of bacterial metabolites is directly involved as well [39]. There are two main alterations that occur between a CRC patient and a normal individual. First, there are alterations in the metabolism of polyamine, and the second is alterations in short-chain fatty acids. (SCFAs), where lower levels of SCFAs are produced, especially butyrate, which is believed to possess anti-tumor properties [39,47].

Polyamines are essential for cell growth; they are aliphatic amines whose metabolism is dysregulated in a CRC patient [39]. The key enzyme of the polyamine biosynthetic pathway, which is known as ornithine decarboxylase (ODC), is expressed at higher levels in a CRC patient compared to a healthy individual [39]. This increase in expression is believed to be associated with the tumorigenesis of CRC [39].

Another important metabolic alteration that is believed to be linked to CRC is the production of lower levels of short-chain fatty acids (SCFAs), more specifically butyrate, as mentioned above [39]. SCFAs are the leading end products of the fermentation of non-digestible carbohydrates [48]. SCFAs are formed through the saccharolytic fermentation of carbohydrates which avoid digestion and are absorbed in the small intestine [48]. Furthermore, as an individual consumes fiber, SCFAs such as propionate, acetate, and butyrate are produced [49]. Of these three SCFAs, butyrate is the most studied, and that is due to its anti-tumor nature [47]. This metabolite can affect the differentiation and growth of colonocytes, hence, having anti-tumor effects and playing a role in the prevention of colorectal cancer [49]. Furthermore, this metabolite is important for maintaining colonic epithelium [48]. Therefore, it is important for individuals to increase fiber intake and, if not possible, then take butyrate supplements; because, in addition to the anti-tumor effects butyrate possesses, it is also shown to improve insulin sensitivity and decrease adiposity [49]. Furthermore, propionate is capable of inducing apoptosis in CRC cell lines, a loss in mitochondrial membrane potential, release of cytochrome c, and generation of ROS [50]. Moreover, propionate causes a decrease in the growth of tumorigenic lesions within the gut, and this is due to its role as an inducer of histone acetylation within CRC cells [50]. Likewise, acetate is also capable of reducing cell viability and inducing apoptosis [50]. One research group has found that treatment of CRC cells with acetate leads to a reduction in cell proliferation and the induction of apoptosis (Table 4) [50].

## 8. Inflammation and the Role of the Gut Microbiome

It is believed that the progression and development of CRC are strongly associated with the gut microbiota of an individual [51]. The development of CRC commonly starts through a sequence of events known as the “adenoma-carcinoma sequence” [52]. Initially, CRC formation commences with the transformation of an individual’s normal epithelium into a hyper-proliferative epithelium [52]. As the cells transform into hyper-proliferative intestinal epithelial cells (IECs), they can form adenomas due to their loss of structure and organization [50]. Furthermore, these adenomas are then capable of growing and invading the submucosa, thus, becoming cancerous and disseminating into the colon [52]. The sequence of events described is highly dependent on various molecular alterations, hence why it can be thought of as “heterogenous” [52]. Although there are various factors that influence CRC formation (such as diet), chronic inflammation is believed to be another major risk associated with it [53].

It has been observed that an individual diagnosed with CRC will have changes in the composition of their gut microbiota. In fact, the gut microbiota can impact the development of cancer; importantly, the colon harbors a high bacterial density within itself [52]. Previously, a basic sequence of events was described, where a normal intestinal cell turns into a hyper-proliferative intestinal cell, which then turns into an adenoma and thus can turn cancerous and spread to the colon [54]. However, to go into further detail, for a normal colonic epithelium to turn cancerous, a series of inflammatory-immunological, as well as genetic factors, need to come to play [54]. Precisely, the inflammatory state of the colon plays a crucial role in the development of CRC [54]. In fact, research has shown that patients suffering from inflammatory bowel disease (IBD) are more prone to developing CRC; the gut microbiome has an essential role in the development of intestinal inflammation, which contributes to CRC and IBD [54,55]. Furthermore, it was also found through various studies conducted on animal models that the use of non-steroid anti-inflammatory drugs reduced the occurrence of CRC [52]. While mice treated with dextran sodium sulfate (DSS), an inflammatory agent, were found to be more susceptible to CRC.

Moreover, the gut microbiota of an individual has the capability to influence the inflammatory state of the colon, thus increasing the risk of CRC [54]. For instance, bacterial stimuli have the capability of activating immune signaling pathways, which in turn results in the loss of homeostasis and the production of an inflammatory environment [54]. As a result of these microbial stimuli, inflammasomes can be activated. Inflammasomes are multiprotein complexes that promote inflammation as a response to cellular distress or pathogens [56]. 

Additionally, microbial stimuli can also result in the activation of the nuclear factor kappa-light-chain-enhancer of activated B cells (NF-κB) pathway [54]. This pathway promotes the production of proinflammatory cytokines such as IL-6. Furthermore, the gut microbiota is also capable of influencing regulatory T cells and Th17—a subset of T helper cells. These cells are capable of modulating inflammation within the colon [54].

Furthermore, studies were conducted, and it was found that in germ-free mice, intestinal tumorigenesis was reduced [54]. Moreover, the gut microbiome in a CRC patient holds pro-and antitumor immune cells as well as various microbes [54]. These microbes have adjusted to the conditions created by the immune and tumor cells [54]. This explains why bacterial genres such as Clostridium and Bacteroides are reduced [54]. These bacteria are incapable of competing with the growing tumor cells and immune cells, which are producing inflammatory compounds toxic to them [54].

## 9. Possible Therapeutic Interventions: Fecal Microbiota Transfer 

Currently, treatment strategies for CRC include surgery, radiation therapy, and chemotherapy; however, each of these treatment strategies possesses various limitations as well as harmful side effects [57]. Currently, chemotherapeutics such as irinotecan, fluorouracil, capecitabine, and oxaliplatin are in use in addition to the immunotherapeutic and biologics such as anti-EFGR, anti-PD-1, anti-VEGF, MEK and BRAF inhibitors [58]. A major issue of the current treatment strategy is the capability of acquiring resistance towards these therapeutics leading to a rise in side effects such as infection [58].

Based on the characteristics of the tumor, such as the number, the progression, and its localization, an appropriate choice of treatment will be planned for the patient [59]. A patient may fall into one of four groups (group 0, group 1, group 2, and group 3) [59]. Group 0 patients have no metastatic disease and lack poor prognostic signs; hence, they are often recommended for surgical treatment [59]. Group 1 patients have resectable metastatic disease, and they are treated with chemotherapy to reduce the number of metastases hence enabling surgery later on [59]. Group 2 patients are those with disseminated unresectable disease. Hence the treatment will only be comforting with its main aim to reduce the symptoms, aggressiveness, and extension of the disease [55]. Finally, group 3 patients will be those with unresectable disease and a lack of intensive treatment; the treatment they will obtain will only be to prevent tumor progression and increase treatment-free life [59].

The imbalance in the gut microbiota of an individual plays a role in the promotion and progression of CRC through various mechanisms, such as inflammation, as explained earlier. Hence why to manage CRC, various therapeutic methods have come to play. The most recent of which is fecal microbiota transplantation (FMT) [60]. This therapeutic method is believed to be the modern way of modulating the gut microbiota [60]. Although plenty of research is still to be performed on the use of FMT in the treatment of colorectal cancer, it is currently widely used to treat resistant *Clostridium difficile* infections (CDI) [61]. Moreover, a method similar to that of FMT was first described by a Chinese scientist about 1700 years ago, where a patient diagnosed with antibiotic-associated diarrhea was treated with fecal enemas [62].

FMT has been shown to be an effective treatment for CDI infections; it is approved by the United States Food and Drug Administration [63,64]. This bacterial infection is believed to be the most common cause of nosocomial diarrhea in the United States [65,66]. Initially, CDI infections were treated with the antibiotic vancomycin; however, later, it was found that more virulent strains of CDI have emerged due to antibiotic resistance [64]. When patients acquire a CDI infection, and are treated with antibiotics, the initial host microbiota is suppressed, and an environment friendly to *C. difficile* spores is created. Hence, the spores germinate, and a vegetative form of the bacteria grows. The greater the antibiotic-resistance *C. difficile* possesses, the greater its toxin production is. However, when the patient is treated with FMT, new microbial communities begin to form. CDI infections are found to decrease the usually dominating *Bacteroidetes* and *Firmicutes*. However, when a patient is infused with healthy fecal material from a donor, the healthy bacteria are found to re-dominate, as is the case in the microbiome of the healthy donor [64,66].

To further explain, FMT is the transfer of fecal material from a healthy individual into a diseased recipient. The fecal content from the donor is instilled in the patient using either nasogastric or colonoscopic incubation [67]. This innovative method has the capability to alter the gut microbiota of an individual [60]. It can introduce disease-free healthy microbial organisms to an altered microbial community, thus restoring the guts microbial homeostasis [68,69]. As explained earlier, individuals diagnosed with CRC have an altered: dysbiotic gut microbiota; hence, their fecal microbiome is also altered [60].

With FMT, a reduction in the activation of pro-carcinogenic, inflammatory, and proliferative pathways is aimed for [60]. However, most of the evidence relating to the efficacy of FMT is on CDI treatment and not CRC [68,69]. Yet, studies were conducted on wild mice and laboratory mice in which colorectal tumorigenesis was induced with dextran sodium sulfate/azoxymethane [69]. In this study, the feces of wild mice were transplanted into the laboratory mice, and an improved host fitness and resistance to colorectal tumorigenesis was found [69]. Unfortunately, more studies are to be conducted to ensure the safety of this procedure, especially in the long term [60]. As emphasized earlier, FMT to help treat CRC patients is still under the study process; however, it is important to note that when testing for the efficacy of FMT, immunocompromised patients are excluded in randomized control trials [70]. This is because immunocompromised patients are believed to have a greater risk of developing complications associated with FMT infection [70]. FMT comes with the risk of introducing pathogens to a patient hence the identification of a suitable donor is crucial; nonetheless, the risk of a patient’s aversion towards the transplant is present [71].

Taking into consideration the importance of FMT and its potential in treating CRC patients, further clinical and in vivo studies utilizing this treatment strategy versus CRC patients to allow for the normalization and approval of this treatment for CRC are needed. Moreover, a less time-consuming and accurate screening method to help find a suitable donor should be developed.

## 10. The Use of Pre/Post Biotics as Therapy

Probiotics are defined to be living organisms that, upon administration in appropriate amounts, provide health benefits to the host [72,73,74]. In fact, they have been used for a very long time, more than a century [73,75]. They possess various health benefits, which has made them a potential candidate for CRC treatment and prevention [57]. These organisms have the capability of downregulating chronic inflammation, improving the diversity of the gut microbiota, and reducing the production of carcinogenic compounds during dysbiosis [39]. Furthermore, as they manipulate the gut microbiota, they exert anti-mutagenic and anti-cancerous activity [60].

Various studies were conducted on the capability of probiotics to inhibit tumorigenesis and their ability to reduce the toxicity of cancer-related therapy [62]. Due to the various limitations current CRC treatment strategies possess, probiotics have now been considered as possible therapeutic drugs [57,62]. It is well known that they can interact with resident microflora, thus giving rise to various host-microbe interactions. Furthermore, it is through these interactions that they can inactivate carcinogens, modulate apoptosis and cell differentiation, and inhibit the tyrosine kinase signaling pathway [57]. Through *in vitro* studies, it has been found that probiotic strains are capable of exerting antimutagenic activity because of their structural peptidoglycans, secretory glycoproteins, and polysaccharides; also because of their production of anticarcinogenic compounds [74]. For example, the probiotic *Lactobacillus casei* was found to produce ferrichrome, an anticarcinogenic compound showing tumor-suppressive effects [57]. This molecule induces apoptosis of the cancer cells through the c-Jun N-terminal kinase pathway [39].

Likewise, probiotics can bind to mutagens which results in biotransformation and detoxification. Additionally, through the interference in several different signaling pathways, apoptosis can be induced. Based on a study conducted, *Propionibacterium acidipropionici* and *Propionibacterium freudenreichii* yield propionate and acetate, which are SCFAs capable of inducing cellular apoptosis in human colorectal cancer cell lines [57]. These probiotic strains were found to activate the caspase 3 enzyme, leading to chromatin condensation, apoptotic nuclei body production, and finally, the production of reactive oxygen species [57]. *Propionibacterium freudenreichii*’s secretion of acetate and propionate leads to the activation of the mitochondrial apoptosis pathway, which in turn kills human colorectal cancer cells [57]. Furthermore, probiotic strains of *Pediococcuspentosaceus* FP3, *Enterococcus faecium*, *Lactobacillus salivarius* FP25, and *L. Salivarius* FP35 were found to show antiproliferative effects against colorectal adenocarcinoma cells [57]. Moreover, the tyrosine kinase signaling pathway, which is responsible for cellular differentiation and proliferation, can be inhibited by probiotics [75]. The receptors found in tyrosine kinases can act as targets for inhibitors and hence can be a potential anti-CRC therapy [75].

Various studies have been conducted to understand the effect probiotics have in relation to tumor growth and CRC. For example, a probiotic containing *Bifidobacterium* was orally administered in mice that contained unfavorable gut microbes. After the administration, an increase in anti-tumor efficacy was found. Hence, tumor growth was abolished [61]. Another study conducted on an animal CRC model administered *Lactobacillus acidophilus* and *Bifidobacterium bifidum* [60]. The results of this study revealed there was an increase in the expression of tumor suppressor microRNA and a decrease in oncogenes.

Furthermore, probiotics may be used as therapeutic methods to help reduce the side effects of anti-cancer therapy [60]. Patients undergoing chemotherapy often struggle with diarrhea; probiotics have been found to reduce the risk of diarrhea. Based on the results of a study conducted, it was found that the administration of *Lactobacillus rhamnosus* GG reduces abdominal discomfort. In addition, probiotics have been proven to be effective against acute diarrhea [73,74]. Based on numerous studies conducted, it has been found that probiotics have the ability to reduce the rate of antibiotic-associated bacteria [73,74]. In a meta-analysis with 25 randomized trials conducted by McFarland, it was revealed that probiotics are capable of significantly reducing diarrhea associated with antibiotics [74]. Moreover, probiotics are believed to be effective against *Clostridium difficile* associated diarrhea, ulcerative colitis, and hepatic encephalopathy; however, it is believed to be ineffective against Crohn’s disease and acute pancreatitis; additionally, their effectiveness against diarrhea caused by viruses is found to be inconsistent [74]. Additionally, after surgical procedures, probiotics are believed to protect the intestinal mucosa barrier of patients [60]. Unfortunately, there are certain regulatory issues facing the distribution of probiotics; as they are not considered to be drugs, they are not regulated by the U.S. Food and Drug Administration [73,74].

Back in the 1980s, it was claimed that certain components of an individual’s diet could promote the growth of certain bacterial strains [76]. These bacterial strains would be beneficial to the host; hence how, another therapeutic method came to rise, known as prebiotics. Prebiotics are defined to being non-digestible food ingredients that stimulate the growth and activity of certain bacteria in the host’s colon, hence, improving their health [72]. All prebiotics are fiber, yet not all fiber is prebiotic [77]. For a food to be considered a prebiotic, it must resist gastric acidity, hydrolysis by enzymes, and absorption by the upper intestinal tract [77]. Furthermore, it must be fermented by the Intestinal microflora and must selectively stimulate the growth of the intestinal bacteria [77]. Food such as asparagus, chicory, leeks, wheat, oats, garlic, artichokes, and onions are prebiotics [77]. These prebiotics are fermented in the colon, hence, leading to changes in the gut microflora [77]. They stimulate the growth of indigenous bacteria, which is physiologically beneficial to the host [77]. One major fermentative product of prebiotics is SCFAs, of the most important is butyrate [76]. SCFAs are essential in the prevention of CRC, as mentioned earlier; they possess many benefits [76].

Synbiotics refer to the combined effects of probiotics and prebiotics [77,78]. They are believed to hold a variety of health benefits for individuals. These include promoting the growth of beneficial bacteria, promoting the stability of intestinal microorganisms, producing SCFAs, lowering intestinal pH, enhancing immune regulation, and blocking bacterial translocation [77]. Researchers believe synbiotics, prebiotics, and probiotics are capable of regulating immune function through the upregulation of anti-inflammatory factors, reducing precancerous lesions, and preventing CRC [77]. A very popularymbioticc is the combination of *Bifidobacterium* or *Lactobacillus* with fructooligosaccharides (FOS) [77]. In symbiotic preparation, the prebiotics and probiotics commonly used are galactose oligosaccharides, xylose oligosaccharides, *S. boulardii*, *Lactobacillus* spp., *Bifidobacteria* spp., and *B. coagulans* [77]. Furthermore, kefir: fermented food acting as synbiotics, plays crucial roles in anti-mutagenic and anti-cancer activities [77]. Kefir has been shown to repair DNA damage and inhibit colon cancer Caco-2 and HT29 cell proliferation [77].

As the popularity of pro, pre and synbiotics is increasing, researchers should study these various biotics and further develop them into approved anticancer treatments widely used globally.

## 11. Concluding Remarks

As colorectal cancer is common and maintains a high mortality rate, it is important to understand the factors leading to this illness [15,16]. The alterations in the gut microbiome play a major role in the progression and development of CRC [40]. Hence why it is important for individuals to maintain a balanced gut microbiome by following a healthy diet consisting of fibers, as they contain SCFAs such as butyrate, which is known for its antitumor properties [39,45]. Nevertheless, alternative treatments such as fecal microbiota transfer, postbiotics, and prebiotics have caught the attention of researchers, especially since available treatments possess various side effects. However, further studies understanding the efficacy of the fecal microbiota transplant should be conducted. Additionally, it is necessary to understand the various microorganisms in the gut microbiome and their contribution to CRC. Moreover, further research studying the metabolic crosstalk between the cancer cells and microbiome within which the tumor is developing can and should be conducted. Finally, as the role of microorganisms is becoming more evident, further studies understanding the mechanisms of actions of each microorganism in carcinogenesis may be performed.

## Figures and Tables

**Table 1:** Various bacterial species are found to associate positively or negatively with CRC. In patients diagnosed with CRC the bacteria families found to have increased in number include *Alistipes*, *Akkermansia* spp., *Fusobacteria*, *Porphyromondaceae*, *Coriobacteridae* and *Methanobacterials*. Whereas the bacteria species belonging to *Bifidobacterium*, *Lactobacillus*, *Ruminococcus*, *Faecalibacterium* spp., *Treponema* and *Roseburia* are decreased in number [27].

Bacterial Species	Increase or Decrease in Colorectal Cancer (CRC) Patients
*Alistipes*	Increase
*Akkermansia* spp.	
*Fusobacteria*	
*Porphyromondaceae*	
*Coriobacteridae*	
*Methanobacterials*	
*Bifidobacterium*	Decrease
*Lactobacillus*	
*Ruminococcus*	
*Faecalibacterium* spp.	
*Treponema*	
*Roseburia*	

**Table 2:** The oral bacteria found within tissue samples of CRC patients include *Fusobacterium*, *Peptostreptococcus*, *Mogibacterium* spp. and *Porphyromona* [32].

Oral Bacteria Associated Found in CRC Patients
*Fusobacterium*
*Peptostreptococcus*
*Mogibacterium* spp.
*Porphyromona*

**Table 3:** The diet an individual follows plays a major role in their chances of developing CRC. Moreover, the diet an individual follows impacts the diversity of microorganisms inhabiting the gut. Alterations in the gut microbiome leads to an increased risk of CRC development.

The Diet an Individual Follows	Impact of the Diet
High fat diet: rich in processed/red meat and high fat dairy products	Higher secondary bile production Insulin Resistance Facilitation of carcinogenesis Leads to an increase in bile-tolerant microorganisms: *Bacteroidetes*, *Bilophila* and *Alistipes* [37,38]
Diet rich in whole grains/high fiber intake	Leads to a decrease in risk of CRC development [39] Increase in *Firmicutes* and *Proteobacteria* [37]
High alcohol ConsumpBtion	Leads to gut dysbiosis [36] Disruption of intestinal epithelial barrier Accumulation of proinflammatory cytokines in intestinal epithelial barrier [14] Increase in number of *Bacteroidetes* and *Verrucomicrobia*. Decrease in number of *Firmicutes*

**Table 4:** The consumption of fiber leads to the production of SCFAs. Of most interest are butyrate, acetate and propionate all three of which hold anti-tumor effects and allow for the reduction in risk of developing CRC.

Short Chain Fatty Acids (SCFAs)	Impact of SCFAs
Butyrate	Affects the differentiation and growth of colonocytes Attains anti-tumor effects [49] Important for maintaining the colonic epithelium [48] Improves insulin sensitivity [49]
Acetate	Reduces cell viability Induces apoptosis [50]
Propionate	Induces apoptosis in CRC cell lines Decreases the growth of tumorigenic lesions within the gut [50]

## Data Availability

Not applicable.
